# Expansion of a Telomeric *FLO/ALS*-Like Sequence Gene Family in *Saccharomycopsis fermentans*

**DOI:** 10.3389/fgene.2018.00536

**Published:** 2018-11-13

**Authors:** Beatrice Bernardi, Yeseren Kayacan, Jürgen Wendland

**Affiliations:** Department of Bioengineering Sciences, Research Group of Microbiology, Functional Yeast Genomics, Faculty of Sciences and Bioengineering Sciences, Vrije Universiteit Brussel, Brussels, Belgium

**Keywords:** flocculation, comparative genomics, telomere, gene family, non-conventional yeast, *FLO8*, *MSS11*

## Abstract

Non-*Saccharomyces* species have been recognized for their beneficial contribution to fermented food and beverages based on their volatile compound formation and their ability to ferment glucose into ethanol. At the end of fermentation brewer’s yeast flocculate which provides an easy means of separation of yeasts from green beer. Flocculation in *Saccharomyces cerevisiae* requires a set of flocculation genes. These *FLO*-genes, *FLO1*, *FLO5*, *FLO9*, *FLO10*, and *FLO11*, are located at telomeres and transcription of these adhesins is regulated by Flo8 and Mss11. Here, we show that *Saccharomycopsis fermentans*, an ascomycete yeast distantly related to *S. cerevisiae*, possesses a very large *FLO/ALS*-like Sequence (*FAS*) family encompassing 34 genes. Fas proteins are variable in size and divergent in sequence and show similarity to the Flo1/5/9 family. Fas proteins show the general build with a signal peptide, an N-terminal carbohydrate binding PA14 domain, a central region differing by the number of repeats and a C-terminus with a consensus sequence for GPI-anchor attachment. Like *FLO* genes in *S. cerevisiae*, *FAS* genes are mostly telomeric with several paralogs at each telomere. We term such genes that share evolutionary conserved telomere localization “*telologs*” and provide several other examples. Adhesin expression in *S. cerevisiae* and filamentation in *Candida albicans* is regulated by Flo8 and Mss11. In *Saccharomycopsis* we identified only a single protein with similarity to Flo8 based on sequence similarity and the presence of a LisH domain.

## Introduction

Non-conventional yeasts have long been studied based on their identification in spontaneous fermentations around the world. Their additional value for fermentation is due to the more complex aroma profiles they produce compared to *Saccharomyces cerevisiae*. This was shown, e.g., for the use of *Torulaspora delbrueckii* and *Saccharomyces bayanus* in bread fermentations (Aslankoohi et al., 2016). The change of the previously dominating view of non-*Saccharomyces* yeasts as spoilage organisms to valuable and increasingly successful contributors to industrial fermentations may best be seen by the introduction of *Brettanomyces* (*Dekkera bruxellensis*) into beer and wine productions (Schifferdecker et al., 2014; Blomqvist and Passoth, 2015; Steensels et al., 2015). Yet, other genera including *Candida*, *Hanseniaspora*, *Kluyveromyces*, *Metschnikowia*, and *Pichia* have been repeatedly identified in biodiversity studies analyzing spontaneous fermentations (Urso et al., 2008; Capozzi et al., 2015). Besides flavor contributions interest also stems from the ability of non-conventional yeasts to reduce the final alcohol content of fermented beverages (Ciani et al., 2016; Rossouw and Bauer, 2016).

In natural fermentations running over weeks diverse successions in the microbial diversity have been observed in coffee fermentations and in the fermentation of Belgian beers (lambic beers or red-brown ales) by autochthonous microorganisms (Silva et al., 2008; Wu et al., 2015; Snauwaert et al., 2016). Improved sequencing technology allows for large scale phylogenomics to explore the biodiversity and successions in such fermentations or to understand the evolution of yeasts in general and of a large variety of *Saccharomyces* strains in particular also with regard to human selection (Illeghems et al., 2012; De Filippis et al., 2017; Sternes et al., 2017; Peter et al., 2018). Taming this biodiversity, i.e., making use of different yeasts in mixed-fermentations, still proves challenging. To be commercially successful, non-conventional yeasts will have to compete with *S. cerevisiae* in terms of fermentation ability, alcohol tolerance, aroma compound formation, and compliance with current process technology.

One central aspect in the processing of fermentations is the ability of brewer’s yeast strains to flocculate at the end of fermentation. This provides a cost-effective means to remove large quantities of yeast from the alcoholic beverage and reuse these yeasts by re-pitching them in a new brew (Stewart, 2018). Flocculation is a reversible aggregation of yeast cells forming large aggregates, flocs, that rapidly sediment – in contrast to settling of cells by gravitational sedimentation – to the bottom of the fermentation medium in bottom fermenting yeasts or float at the top in top fermenting yeasts. Aggregation is cation, mainly Ca^2+^, dependent and flocs can be dispersed by the addition of EDTA (ethylenediaminetetraacetic acid) in *S. cerevisiae* and related strains used in fermentations (Stratford, 1989). Initiation of flocculation occurs at the end of fermentation when carbon or nitrogen sources are depleted and requires the synthesis of Flo proteins. In *S. cerevisiae* flocculation I positively regulated by the cAMP-protein kinase A pathway, MAP kinase signaling via Kss1 and repressed by Ssn6/Tup1 (Verstrepen et al., 2003; Verstrepen and Klis, 2006). The main transcription factor inducing the expression of *FLO* genes, however, is Flo8, which can dimerize with another protein of similar domain structure, Mss11. Laboratory yeast strains derived from S288C are non-flocculent as they harbor a nonsense mutation in *FLO8* at codon 142 converting a tryptophan codon into a stop codon. Flo8 activates the expression of *FLO* genes including the *FLO1/5/9* adhesin family as well as *FLO11*. *FLO*-genes of *S. cerevisiae* are located near the telomeres of different chromosomes and tend to show genetic instability by changes in size, mainly of the repeat length, e.g., induced by recombination between different telomeres (Carro et al., 2003).

Adhesins are ubiquitous cell surface proteins which facilitate cell–cell adhesion or cell-surface adherence. Not surprisingly, use of adhesion molecules serves different lifestyles, e.g., cell–cell attachment and biofilm formation on the one hand, but virulence and infection on the other. Fimbriae (thin appendages) of gram-negative bacteria act as adhesins, e.g., by themselves or by expressing a minor adhesin component located at the fimbrial tip. Fimbriae bind to carbohydrate residues, e.g., the adhesin FimH to D-mannose (Klemm and Schembri, 2000). Carbohydrate binding proteins, i.e., lectins, are ubiquitous in nature and occur in plants, animals, and fungi (and even viruses) (Boyd and Shapleigh, 1954; Sharon and Lis, 2004; Hassan et al., 2015; Hirabayashi et al., 2015). With genomics discovery of many more lectin genes a protein family-based classification of lectins – instead of a carbohydrate-binding specificity-based classification – was introduced (reviewed by Finn et al., 2014). In the homobasidiomycete *Coprinopsis cinerea* three galectins (encoded by *CGL1-3*) are expressed in the fruit bodies (Boulianne et al., 2000; Wälti et al., 2008). Mutations in the carbohydrate binding site of lectins may alter their carbohydrate binding specificity (Hassan et al., 2015).

In ascomycetes the adhesins from *Candida albicans* (*ALS* genes) and *S. cerevisiae* (*FLO* genes) are by far the best characterized (for details on other ascomycete adhesins see Lipke, 2018). Yet, most adhesins have a similar domain structure: N-terminal secretion signal peptide, a conserved N-term with ligand binding domain which is crucial for the functional diversity of adhesins, a central repeat domain that may be highly N- and O-glycosylated and a C-terminal domain that allows the addition of a GPI-anchor and thus the covalent attachment to the cell wall (Lipke, 2018). This offers facile ways to use bioinformatics data mining for the discovery novel adhesins. Adhesins differ by the length and sequence of their internal repeats. Generation of length polymorphisms may occur via DNA-replication errors or due to unequal sister chromatid exchanges (Verstrepen and Fink, 2009). In *C. albicans* and *C. glabrata* adhesins are termed agglutinin-like sequence (ALS) and epithelial adhesins (EPA), respectively (Gabaldon et al., 2013; Hoyer and Cota, 2016). For *C. albicans* other adhesin families have been described, namely the HWP and HYR families (De Groot et al., 2013) and in *S. cerevisiae* Aga1 and Fig2 are adhesins involved in mating and biofilm formation (Brückner and Mösch, 2012).

Fungal adhesins mediate contact interactions of cells with the environment. This can be for “social” behavior, e.g., in mating, colony and/or fruit body formation and biofilm formation or for “aggressive” behavior mediating host–pathogen interactions as seen in the human pathogens *C. glabrata* and *C. albicans* (Dranginis et al., 2007). *Saccharomycopsis* species have been described as fungal necrotrophs that kill other fungi via penetration pegs (Lachance and Pang, 1997). They may therefore have a dual use for their adhesins as they could employ them for flocculation at the end of fermentation and/or for attaching to fungal prey cells at the onset of their attack.

Non-conventional yeasts may introduce new flavors to alcoholic beverage fermentation but should conform with current process technology. *Saccharomycopsis* yeasts, more closely related to *Wickerhamomyces* and *Ascoidea* species than to *S. cerevisiae*, have previously been found in spontaneous fermentations. *S. fibuligera* and *S. fermentans*, for example, were found in rice wine or palm wine fermentations (Ouoba et al., 2012; Kurtzman and Robnett, 2013; Carroll et al., 2017; Farh et al., 2017). This demonstrates that this genus harbors suitable and experienced strains for alcoholic beverage fermentation and thus warrants further analysis. For *S. fibuligera* a whole genome sequence analysis has been published indicating a gene repertoire for starch degradation; additionally, a hybridization event between two closely related species has been discovered (Choo et al., 2016). We recently presented a draft genome sequence of *S. fermentans*, which we now analyze in more detail focussing on the *FAS* gene family (Hesselbart et al., 2018).

Interestingly, we found an amplification of the *FAS* gene family at *S. fermentans* telomeres in a similar manner previously observed in *S. cerevisiae*. We termed those orthologous or paralogous genes that share evolutionarily conserved positions at telomeres “**telologs**” and identified several additional telologs between *S. cerevisiae* and *S. fermentans*. The Fas family of *S. fermentans* was compared to *S. cerevisiae* Flo proteins and *C. albicans* Als proteins. Additionally, a gene with similarity to the *FLO8/MSS11* transcription factors was identified that may be instrumental in regulating this gene set for flocculation at the end of fermentation. Furthermore, cell–cell adhesion may be a key element in initiating necrotrophic mycoparasitism, the ability of *S. fermentans* to penetrate and kill prey fungi to acquire their nutrients.

## Materials and Methods

### Strains and Culture Conditions

*Saccharomycopsis fermentans* (CBS 7830, wild type, heterothallic) and the lager yeast strain Weihenstephan WS34/70 (allotetraploid) were grown in rich medium (YPD; 1% yeast extract, 2% bacto peptone, 2–20% glucose) at 30°C. Mat formation was assayed on low agar YPD plates containing 0.3% agar as described previously (Reynolds and Fink, 2001; Cullen, 2015). The culture for the flocculation assay was prepared by inoculating 50 mL YPD with 500 μL of either *S. fermentans* or WS34/70 from a water stock in a 250 mL Erlenmeyer flask. The cultures were incubated at 30°C in a rotary shaker with 150 rpm for 48 h. The flocculation test was done in the following way: 5 mL of each yeast culture was diluted with 5 mL of YPD and placed in a glass tube. The samples were rigorously vortexed and then placed vertically in a stand to monitor flocculation. To test if flocculation was calcium dependent EDTA (ethylenediaminetetraacetic acid, 50 mM final concentration) was added.

### Draft Genome Sequencing and Assembly

Draft genome sequencing and the assembly strategy of the *S. fermentans* genome was recently published (Hesselbart et al., 2018). The genome sequence is available at GenBank under accession number JNFW00000000. The *FAS* genes are listed in Supplementary Table S1.

### Gene and Protein Bioinformatic Analyses

A more detailed comparative genomic analysis of the *S. fermentans* genome will be published elsewhere. The scaffolds of the *Saccharomycopsis* genome were compared against the *Saccharomyces* Genome Database^1^ using BLAST tools (available at http://blast.ncbi.nlm.nih.gov). This identified the set of Fas proteins based on their sequence similarity to *S. cerevisiae* the *FLO1/5/9* family. Fas proteins were further analyzed using several webtools with default settings as follows: for the presence of signal peptides the SignalP 4.1. server at http://www.cbs.dtu.dk/services/SignalP/ was used. The PA14 domain was identified using the NCBI conserved domain tool available at https://www.ncbi.nlm.nih.gov/Structure/bwrpsb/bwrpsb.cgi. GPI anchors and omega site predictions were done at http://gpcr.biocomp.unibo.it/predgpi/. Repeats sequences of central domains of Fas proteins were identified using RADAR^2^. The individual repeats of all Fas genes were extracted (488 in total) and aligned using MegAlign of the DNA Lasergene 15 software package. The multiple sequence alignment generated with MegAlign was used as input sequence alignment for weblogo at https://weblogo.berkeley.edu/examples.html. This generated a consensus sequence for the internal repeats of the Fas proteins. The Flo proteins of *S. cerevisiae* were retrieved from SGD and the Als proteins of *C. albicans* from the *Candida* Genome Database^3^. Consensus sequences for the internal repeats of Flo proteins and Als proteins were processed in the same way as described above for Fas proteins. A sequence distance table comparing protein sequence identities of fungal adhesins was generated by with MegAlign (Supplementary Figure S1). To generate a dendrogram of fungal adhesins full length adhesins from *S. fermentans*, *S. cerevisiae*, and *C. albicans* were aligned using MegAlign. Bootstrapping was performed on the alignment using standard settings and 1000 replicas.

### *FLO8* Identification and Alignment

The *S. fermentans* genome provides a weak hit against either *S. cerevisiae* Flo8 or Mss11 (sequences obtained from the *Saccharomyces* genome database; see text footnote 1). Sequences of other yeast species similar to ScFlo8 and ScMss11 (see text footnote 1) were retrieved form NCBI and used in additional genome wide blast searches^4^ or in alignments (done with MegAlign using default settings). These include *Ashbya gossypii* (AFL194W and AGL300C^5^); *C. albicans* SC5314 (Flo8, C6_04350cp_a; Mss11, CR_04840C_A); *Cyberlindnera jadinii* (CEP23573); *Eremothecium cymbalariae* DBVPG#7215 (Flo8, XP_003647814 and Mss11, XP_003647183); *Lachancea thermotolerans* CBS6340 (Flo8, XP_002552501; Mss11, XP_002554632); *Saccharomycopsis crataegensis* CBS 6447 (JNFX00000000), *S. fodiens* CBS 8332 (JNFV00000000); *Wickerhamomyces anomalus* NRRL Y-366-8 (Flo8, XP_019036814.1; Mss11 XP_019038767); and *Wickerhamomyces ciferrii* (Flo8, XP_011273448; Mss11, XP_011275532).

Dendrograms are based on full length protein alignments and bootstrapping was done with 1000 replicas. For the identification of LisH domain containing proteins in *S. fermentans* all non-overlapping translated ORF sequences were searched at NCBI using the pfam database^6^ with default settings.

## Results

### Mat Formation and Ca^2+^-Dependent Flocculation in *Saccharomycopsis fermentans*

**FIGURE 1:**
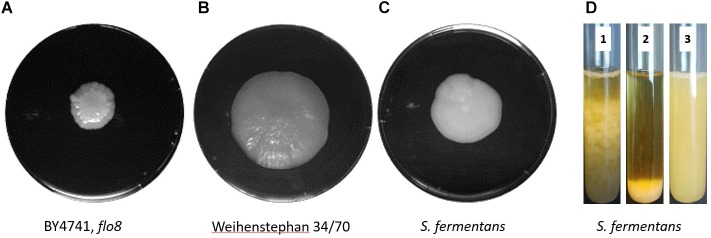
Mat formation in yeast. *S. cerevisiae* BY4741, *flo8* **(A)**, the lager yeast *Weihenstephan* 34/70 **(B)**, and *S. fermentans* **(C)** were grown on low-rigidity 0.3% agar YPD plates for 1 week at 25°C prior to photography. **(D)** Flocculation of *S. fermentans* in liquid media. The same culture was used to indicate speed of flocculation at time point zero, when stirring stopped (1, left) and after 60 s (2, middle). Then after the addition of EDTA to a final concentration of 50 mM after 60 s (3, right) indicating a block in flocculation.

We observed flocculation in *S. fermentans* cultures at the end of the growth phase. To compare the ability to form biofilms we used a mat formation assay as described previously (Reynolds and Fink, 2001; Cullen, 2015). We compared growth of *S. fermentans* with the non-flocculating laboratory yeast strain BY4741 and the lager yeast *Weihenstephan* 34/70. On low rigidity agar plates (0.3%) *S. fermentans* mat formation was not covering an area as large as that of the lager yeast strain but was substantially more spread out than that of BY4741 (Figures 1A–C). *S. fermentans* cultures were grown into stationary phase and flocculation was monitored over a short time interval. In *S. fermentans* flocculation is much faster than sedimentation of cells by gravity with the result that after 1 min cells formed a pellet at the bottom of the test tube (Figure 1D). One of the hall marks of flocculation is its dependency on Ca^2+^ cations. Flocculation can thus be inhibited by the addition of ion chelating molecules such as EDTA (Soares, 2011). Also, in *S. fermentans* flocculation is abolished in the presence of EDTA indicating that *S. fermentans* employs a similar mechanism to generate yeast flocs as brewer’s yeasts (Figure 1D).

### Identification of the *S. fermentans FAS* Gene Family

**FIGURE 2:**
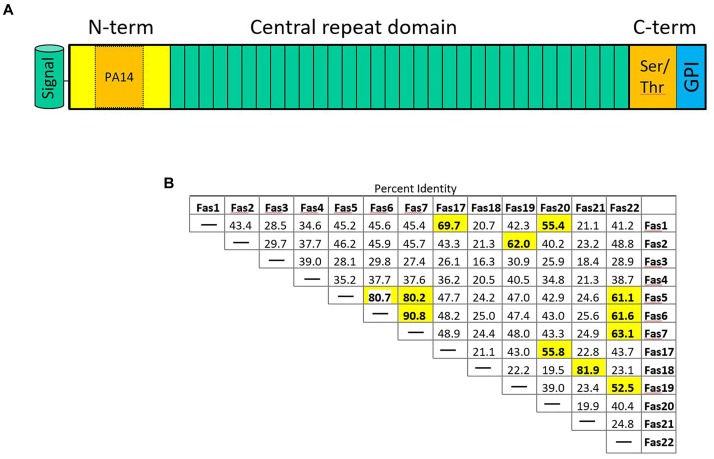
**(A)** Schematic representation of fungal adhesins. The N-terminal signal peptide is followed by an N-terminal domain that contains the PA14 domain promoting protein–carbohydrate interaction. The central domain contains a variable number of repeats (3–36 in *S. fermentans*). The C-terminal domain is rich in Ser/Thr residues and harbors an omega site for GPI-anchor attachment. **(B)** Sequence distance table indicating pair-wise protein sequence identities of the indicated Fas proteins whose coding genes are located on *S. fermentans* chromosome 1 and 4. Sequence identities >50% are highlighted in bold print.

Flocculation genes in *S. cerevisiae* belong to several classes. Flocculation itself is defined as the asexual aggregation of cells into flocs (Stratford, 1989; Bony et al., 1997). Thus, sexual agglutinins, e.g., encoded by *AGA1* and *SAG1*, that in *S. cerevisiae* promote cell to cell adhesion during mating will not be further discussed here. In *S. cerevisiae* the *FLO1/5/9* gene family is distinct from two other flocculins encoded by *FLO10* and *FLO11*. Yet, all *S. cerevisiae FLO* genes show the canonical domain architecture (Figure 2A). This includes an N-terminal signal peptide is followed by a PA14 domain – the name of this domain is derived from “protective antigen” a bacterial toxin found in *Bacillus anthracis* (Rigden et al., 2004). This domain is not only found in adhesins but also in glycosidases and glycosyltransferases consistent with a function in carbohydrate binding (Goossens and Willaert, 2010). In adhesins the PA14 motif makes up a part of the N-terminal domain, which is followed by a central domain of various length consisting of similar sized repeats. The C-terminal domain may bear sites for *O*- and *N*-glycosylation and harbors a recognition site for the addition of a glycosyl-phosphatidylinositol (GPI) anchor with which adhesins are inserted into the cell wall.

*Saccharomycopsis fermentans FAS* genes were identified by blast searches showing highest sequence similarities in their N-termini with S. cerevisiae *FLO* genes. In total 34 *FAS* genes were identified (Supplementary Table S1). While the *FLO1/5/9* family members show more than 85% identity on the amino acid sequence level Fas proteins identified in *S. fermentans* are much more divergent. Only a few Fas protein pairs show more than 90% sequence identity over the entire lengths of their proteins: this includes Fas5/Fas10, Fas6/Fas7, Fas8/Fas28, Fas 14/Fas29, and Fas15/Fas30 (Figure 2B and Supplementary Figure S1). These gene pairs may have evolved more “recently” by gene duplication. *FAS6* and *FAS7* are directly adjacent, while the other gene pairs may subsequently have been transferred to other loci, e.g., by reciprocal translocations (Nag et al., 2004). Several protein pairs show a high sequence identity over their N-term. This adds Fas21/Fas23, Fas9/pseudoFas31, and Fas22/Fas33 to previous set. A large size variation can be found with the smallest *FAS* gene, *FAS27*, coding for a 591 aa protein and the largest, *FAS12*, encoding a protein of 1723 aa. There are variable length C-termini in Fas proteins that allow for differential glycosylation patterns. Amongst the 34 *FAS* genes three may be classified as pseudogenes. These are *FAS3*, *FAS18*, and *FAS31*. This is due to either lack of a signal peptide or a lacking start codon in the latter. The other *FAS* genes conform to the standard domain structure of adhesins with a ∼20 aa signal peptide, the N-terminal domain including the PA14 domain, the central repeat rich domain and the C-terminal domain with omega sites required for GPI-anchoring. The repetitive sequences within *FAS* genes may cause recombination events between closely related sequences, i.e., either within the same gene, between *FAS* genes or between a *FAS* gene and a pseudogene, and thus generate size variations leading to an increase or decrease in gene sizes as described for *S. cerevisiae* (Verstrepen et al., 2005).

### Analysis of the Repeat Structure of *S. fermentans* Fas Proteins

**FIGURE 3:**
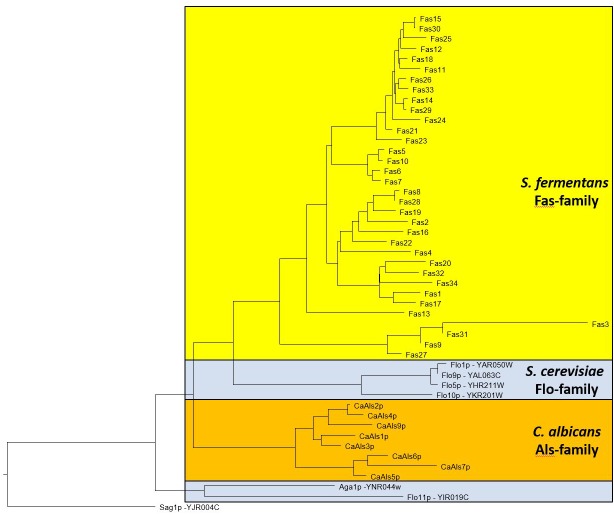
Dendrogram showing relationships between *S. fermentans*, *S. cerevisiae*, and *C. albicans* adhesins. Proteins were aligned using ClustalW and a tree based on this alignment is presented.

**FIGURE 4:**
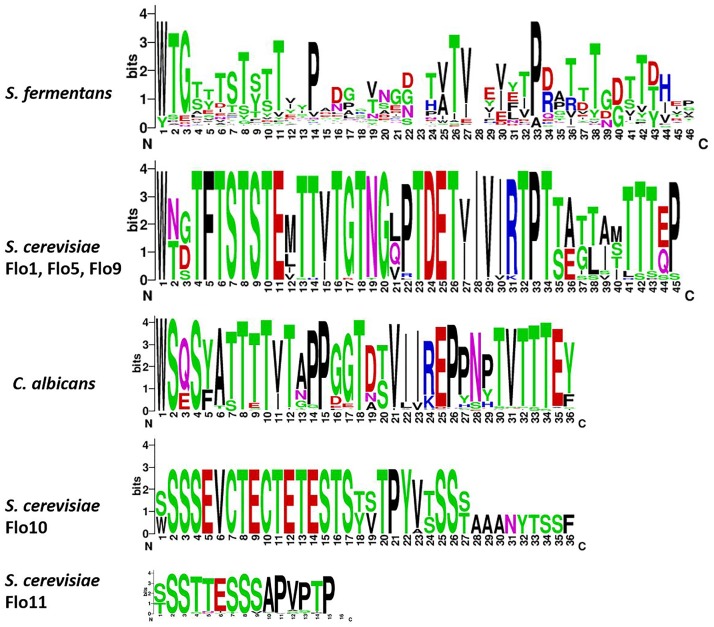
Sequence enrichment plots of adhesin repeats. For the *S. fermentans* and *C. albicans* all repeats of Fas and Als proteins, respectively, were combined. For *S. cerevisiae* the Flo1, Flo5, and Flo9 repeats were combined and Flo10 and Flo11 repeats were treated separately. Repeats were trimmed and aligned using ClustalW and then sequence enrichment plots were calculated using weblogo.

The large size differences in Fas proteins is, mainly due to the various number of repeats in the central region. For example, Fas27 has only 3 repeats while Fas12 encodes 36 repeats while on average 14 repeats can be found. We aligned the different adhesins and a dendrogram indicates that adhesins from *S. fermentans*, *C. albicans*, and *S. cerevisiae* form distinct groups (Figure 3). We then went on and examined the repeat sequences of *S. fermentans* Fas proteins and compared them with the repeats found in *S. cerevisiae* Flo proteins and *C. albicans* Als proteins. In total we identified 488 repeats in Fas proteins (see Supplementary Table S1), 39 repeats in Flo1, Flo5, Flo9; 10 repeats in Flo10; 41 repeats in Flo11 and 140 in the Als proteins, respectively. These repeats were trimmed, aligned and consensus sequences were established using weblogo (see section “Materials and Methods”). Flo10 and Flo11 harbor distinct repeats different from each other and from the Flo1/5/9 family. In Flo10 there are two variants a 27 aa and an extended 36 aa repeat adding an invariant 9 aa sequence of AAANYTSSF to 4 of the repeats. Similarly, Flo11 has a basic 12 aa repeat which, in half of the repeats, is extended by the tripeptide PTP (Figure 4).

In Als proteins the repeat length is uniformly 36 aa with a large degree of sequence identity. The repeats within the Flo1/5/9 family are also highly conserved and are 45 aa in length. Repeats in the Fas proteins are on average 36 aa long. However, there is substantial divergence and all repeats align into a 46 aa consensus sequence. Comparison of the consensus sequences shows a high content of Ser/Thr residues and also conserved Pro residues with the repeats (Figure 4).

### *S. fermentans FAS* Genes Constitute a Telomeric Gene Family

**FIGURE 5:**
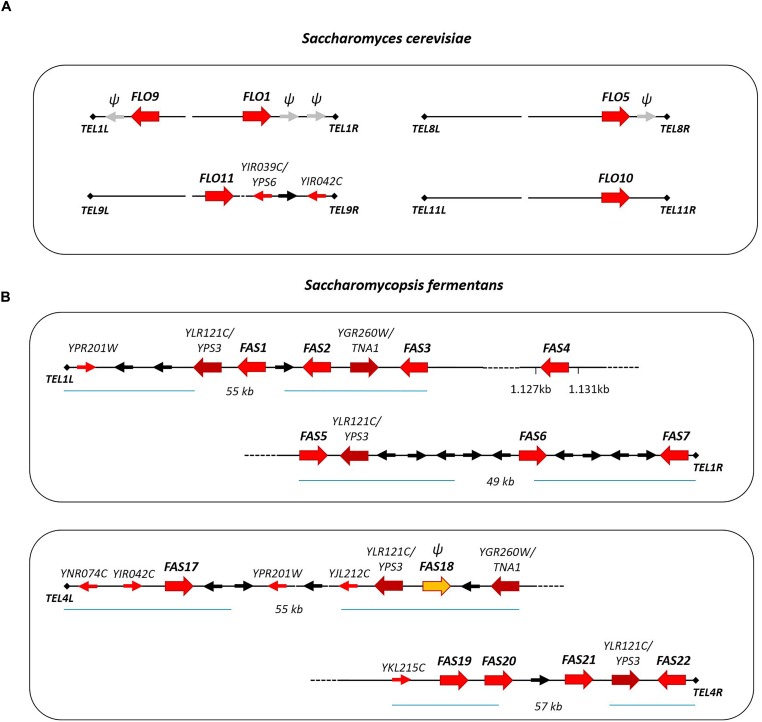
Schematic presentation of adhesin gene localization at telomeric ends of *S. cerevisiae* chromosomes **(A)** and *S. fermentans* chromosomes **(B)**. Adhesin genes are highlighted with large red arrows. Other *S. fermentans* genes belonging to gene families (YPS and TNA) are also represented by large arrows. Genes present at telomere loci in both *S. cerevisiae* and *S. fermentans*, so called telologs, are shown as small red arrows. Additional genes are drawn as black arrows and gray arrows with “ψ” mark *FLO*-like pseudogenes and *FAS18*.

In *S. cerevisiae* several gene families are located at subtelomeric regions including the *FLO* genes (Teunissen and Steensma, 1995; Luo and Van Vuuren, 2009). The *FLO* genes can be found at five different telomeres belonging to four chromosomes. Also, pseudogenes of *FLO*-like sequences were found, e.g., on chromosome I (Bussey et al., 1995; Figure 5A). We have analyzed the location of *FAS* genes in *S. fermentans*. Two chromosomal scaffolds corresponding to *S. fermentans* chromosome 1 and chromosome 4 were analyzed in detail (Figure 5B). At *TEL1R* and *TEL4R* a sequence repeat corresponding to TG_3_(GA)_2-4_ was found and at *TEL1L* a transposon marks the end of the scaffold sequence. Interestingly, each of these chromosomes harbor several *FAS* genes at their telomeric ends. Sequence identity between these Fas proteins is much lower than in the *S. cerevisiae* and *C. albicans* adhesins except for the right arm of chromosome 1. Here Fas5 has 80% identity with Fas6 and Fas7, while Fas6 and Fas7 even share 90% aa sequence identity (see Figure 2B). Most of the *FAS* genes are apparently located at (sub)-telomeric regions, with at least one exception: *FAS4* was found to be at an internal position on chromosome 1.

The *S. fermentans* genome was found to contain several gene families, including the *FAS* genes but also proteases and chitinases and transporters (Hesselbart et al., 2018). Within these expanded gene families, we could identify 26 aspartic proteases encoded by homologs of either YLR120C/*YPS1* (5 genes) or YLR121C/*YPS3* (21 genes) in *S. cerevisiae*, 22 homologs of a *S. cerevisiae* chitinase encoded by YLR186C/*CTS1* and, for example, 10 homologs of the YGR260W/*TNA1* high affinity nicotinic acid permease. Here we found that protease paralogs of *S. cerevisiae YPS3/YLR121C* and genes coding for nicotinic acid permeases, paralogs of *TNA1/YGR260W*, are also located at telomere ends. *YPS3* genes were present at all four telomeres and *TNA1* genes were found at two of these telomeres (Figure 5).

Additionally, at these *S. fermentans* telomeres several homologs of *S. cerevisiae* genes were found that in *S. cerevisiae* are also located in sub-telomeric regions. These include *YIR042C* of unknown function; *OPT1/YJL212C*, an oligopeptide transporter; *OXP1/YKL215C*, a 5-oxoprolinase; *AIP1/YNR074C*, a homolog of the mammalian Apoptosis-Inducing Factor; and *ARR3/YPR201W*, a transporter required for resistance to arsenic compounds (Figure 5). These genes represent telologs, i.e., genes sharing a conserved telomer localization – yet not necessarily at the ancestral locus. Due to the evolutionary distance of the genera *Saccharomycopsis* and *Saccharomyces* it may be expected that these genes kept their telomeric position also in other genera and thus may be useful in genome assemblies.

### Identification of *FLO8* in *Saccharomycopsis fermentans*

**FIGURE 6:**
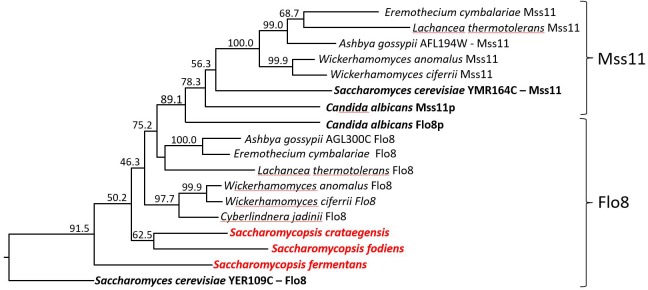
Dendrogram of Flo8 and Mss11 orthologs from different yeast species. Tree showing the positioning of *Saccharomycopsis* Flo8 proteins with other LisH-domain containing proteins from diverse yeast genera. Protein sequences were obtained via NCBI, aligned with ClustalW and the numbering indicates bootstrap values obtained with 1000 replicas. The Mss11 and Flo8 groups are indicated.

Flo8 is a transcription factor that regulates the expression of *S. cerevisiae FLO* genes (Goossens and Willaert, 2010). *C. albicans FLO8* is essential for hyphal morphogenesis and is required for the expression of *ALS1* (Cao et al., 2006). In *S. cerevisiae* and *C. albicans* a second gene, *MSS11*, is also involved in expression of adhesins, together with *FLO8* (Bester et al., 2006; Su et al., 2009). Flo8 and Mss11 contain N-terminal LisH domains (pfam08513) and CaMss11 was shown to interact with CaFlo8 via this domain (Su et al., 2009). We performed BLASTp searches querying the translated ORF-datasets of *S. fermentans*, *S. crataegensis*, and *S. fodiens* for Flo8/Mss11 sequences. The best hit was derived from *Wickerhamomyces ciferrii* (4.3e-023) Flo8p as queries. We aligned Flo8/Mss11 sequences of the three *Saccharomycopsis* species with Flo8 and Mss11 orthologs from other yeasts (Figure 6). The deduced tree indicates a well supported separation between Mss11 and Flo8 proteins and a thus a placement of the *Saccharomycopsis* protein sequences with fungal Flo8 proteins. However, the protein sequences are highly divergent, and similarities are confined to a small N-terminal region.

The key domain of *S. cerevisiae* and *C. albicans* Flo8 and Mss11 proteins is a LisH domain. This allowed an independent search approach from blastp searches. Therefore, we used all non-overlapping translated ORFs from the draft genome sequence of *S. fermentans* and searched the conserved domain pfam database for LisH-domain containing proteins. Only two hits were retrieved, one to Flo8 (*e*-value 5.0e-05, pfam08513) and a second one to a *S. cerevisiae* homolog of Sif2 (*e*-value 2.2e-06, pfam08513), which is known to harbor a LisH domain also in *S. cerevisiae*. This suggests that in *S. fermentans* only one protein corresponding to Flo8/Mss11 is present, which we named *FLO8*. Similarly, only one gene coding for Flo8 was found in *S. fibuligera*, *S. fodiens*, and *S. crataegensis* suggesting that this finding may not be due to the incompleteness of the draft genome sequences even though the closely related *Wickerhamomyces* genus harbors *FLO8* and *MSS11* genes.

**FIGURE 7:**
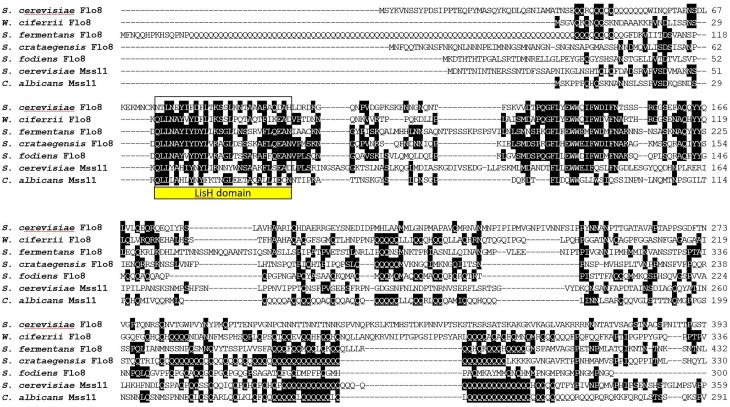
Alignment of LisH-domaining proteins from the indicated fungi. Only the N-terminal part of the alignment is shown. The LisH dimerization domain is boxed. Proteins were aligned using ClustalW and two or more matching residues for each position in the alignment are shaded in black.

An alignment of N-terminal sequences of Flo8 and Mss11 proteins shows the similarity of these proteins in the region encompassing the LisH-domain (Figure 7). In Flo8 and Mss11 glutamine rich regions can be found. These are mostly internal. However, *S. fermentans* Flo8 shows an extended N-terminal region with an enlarged poly-Q-repeat of 89 residues (Figure 7).

## Discussion

Alcoholic beverages such as wine and beer have been produced for several millennia and today the wine and beer sectors constitute key industries in world-wide beverage production. Brewer’s yeasts have been the workhorses for these industries and besides their ability for alcoholic fermentation and flavor production their flocculation at the end of fermentation is most convenient to separate yeast slurries from the produced beverage (Verstrepen et al., 2003). With the craft beer movement came further challenges and innovations in the beverage producing sectors. One is the search for non-conventional, i.e., non-*Saccharomyces*, yeasts to generate more diversity and richness in flavor production. The other is the requirement for strains to be compatible with *S. cerevisiae*, e.g., in co-fermentations, but also with existing brewing technology. Here flocculation plays a major role.

The molecular mechanism of flocculation has been studied for decades and excellent reviews provide detailed insight (Goossens and Willaert, 2010; Soares, 2011; Lipke, 2018). The hall mark of flocculation is based on Flo protein–carbohydrate (mannose) interaction between yeast cells. *S. cerevisiae* harbors different adhesins and particularly the *FLO1/5/9* family is promoting flocculation, while *FLO11* regulates pseudohyphal and invasive growth and sexual adhesins are expressed during mating of haploid yeast cells (Erdman et al., 1998; Lo and Dranginis, 1998). Flocculation occurs in vegetative cells and is calcium-dependent (Stratford, 1989). In lager yeasts this results in the drop-out of flocs to the bottom of the fermentation vessel from where they efficiently can be collected to initiate a new fermentation. In other fungal systems, adhesins promote fungal virulence, e.g., in *C. albicans* or *C. glabrata* (Sui et al., 2017; Lopez-Fuentes et al., 2018).

In *S. fermentans* only paralogs to the *FLO1/5/9* family of *S. cerevisiae* were found, but not *FLO10* and *FLO11*. A comparison of the *FAS* gene family of *S. fermentans* with the *FLO/ALS* gene families in *S. cerevisiae* and *C. albicans* shows that this gene family is much larger in *S. fermentans* and consists of 34 genes (including three potential pseudogenes). A large degree of copy number variation (CNV) has recently been reported in *S. cerevisiae* wine strains. This particularly involved telomeric gene families and includes *FLO* genes and hexose transporters of the *HXT*-family, but also genes involved in copper resistance (Steenwyk and Rokas, 2017). This diversity found in wine yeasts may, of course, be the result of human selection and the yeasts’ adaptation to different fermentation environments. Besides CNV, there are also substantial size differences between adhesin proteins which are largely due to the number of internal tandem repeats. While individual tandem repeats in Flo-proteins and Als-proteins are quite highly conserved in sequence, Fas proteins harbor a larger degree of divergence with only a third of the residues within these repeats being highly conserved. Several of the Fas proteins (namely Fas1,6,7,8,12,17,20,25,28,32) harbor a single RK-dibasic motif in the repeat region, while the KK-motif is found four times in Fas16 and once each in Fas25 and Fas32 and the KR sequence is found once each in Fas20 and Fas32. Such dibasic motifs may serve as proteolytic cleavage sites by aspartic proteases, e.g., the *S. cerevisiae* yapsins or the *C. albicans* Sap9/Sap10 proteases (Schild et al., 2011). In *S. fermentans* there is a large family of aspartic proteases available whose genes are also localized at telomeres like the *FAS* genes (see Figure 5).

These conserved residues with the central repeats may be involved in O-linked glycosylation and, in case of the conserved prolines, for structural purposes to establish rod-like structures (Jentoft, 1990). *S. fermentans* is strongly flocculant at the end of fermentation. This flocculation can be abolished by sequestration of Ca^2+^ ions by EDTA indicating a closely related flocculation mechanism compared to *S. cerevisiae* (Verstrepen and Klis, 2006).

One of the striking physiological features of *Saccharomycopsis* species is their predacious behavior. *Saccharomycopsis* species are auxotrophic for organic sulfur compounds and, e.g., upon starvation for methionine generate penetration pegs and kill fungal prey cells (Lachance et al., 2000). As one of the initial steps, cell–cell attachment could play a key role toward successful predation. However, how *S. fermentans* differentiates self from non-self to generate either flocs or initiate predation is currently unknown. In three *Saccharomycopsis* species, *S. fermentans*, *S. fodiens*, and *S. crataegensis*, several large gene families have been identified through draft-genome sequencing. This includes, the *FAS* genes, aspartic proteases (paralogs of *S. cerevisiae YLR120C/YPS1*-*YLR121C/YPS3*), chitinases (similar to *YLR286C/CTS1*), and transporters (*YGR260W/TNA1* in *S. cerevisiae*). This suggests gene family evolution supported predacious behavior in *Saccharomycopsis*. Strikingly, the placement of several of these gene families, and particularly of the *FAS* and yapsins genes, at telomeric regions in *S. fermentans* resembles the evolution of gene families at *S. cerevisiae* telomeres. Similar amplifications of genes at subtelomeric regions were also found for aspartic protease (*SAP*) genes in *C. albicans* and chitinases in the mycoparasite *Trichoderma reesei* (Naglik et al., 2003; Liti and Louis, 2005; Seidl et al., 2005).

Due to the plasticity of telomeres, efforts to reconstruct ancestral gene orders at these positions are intrinsically difficult (Liti and Louis, 2005). When reconstructing the ancestral genome of yeast prior to the Whole Genome Duplication telomeric regions encompassing the terminal 10 genes could not be assigned to a single chromosome due to the fast turnover particularly within telomeres (Gordon et al., 2009). Here our analysis with *S. fermentans* shows that evolution at telomeres may have led to gene family expansions and relocation of ancestral telomeric genes. Remarkably, six genes that are present at *S. cerevisiae* telomeres were also found at telomeres in *S. fermentans*. For these genes we introduce the term “telologs,” i.e., paralogs located at telomeric positions. This further suggests that phylogenomics of a sufficient amount of complete yeast genomes will eventually determine the telomere gene set of the yeast ancestor.

Flocculation genes are controlled by several mechanisms, including telomere silencing, epigenetic regulation, the cAMP-dependent protein kinase A pathway, a MAP kinase pathway, negatively by Sfl1 and positively by Flo8 and Mss11 (Teunissen et al., 1995; Kobayashi et al., 1996; Halme et al., 2004; Bester et al., 2006; Verstrepen and Klis, 2006; Fichtner et al., 2007). We have identified two *SFL1* paralogs in *S. fermentans*, one on chromosome 1 and another on chromosome 4, as well as in *S. fodiens* and *S. crataegensis*. On the other hand, extensive searches for *FLO8* and *MSS11* suggested that predator yeasts only contain one ortholog of a *FLO8*-like transcription factor best recognized by its LisH domain. This domain is part of the LUFS domain that is also conserved in *Arabidopsis thaliana LUG* (*Leunig*) and mouse *LIS1* (Kim et al., 2004; Shrestha et al., 2014). Additionally, also in *S. fibuligera* only one *FLO8* gene is present (Choo et al., 2016). Functional analysis of *S. fermentans FLO8* will determine its role in the expression of *FAS* genes and/or in predation. In *S. cerevisiae* and *C. albicans* Flo8 and Mss11 form heterodimers via their LisH-domains, while both Flo8 and Mss11 of *C. albicans* can also form homodimers (Kim et al., 2014). Overexpression of *CaFLO8* can suppress the *mss11* deletion but *MSS11* overexpression failed to rescue the hyphal growth defect of *flo8* in *C. albicans* (Su et al., 2009). This indicates a more important role of *FLO8* and may provide an explanation for the loss of *MSS11* in *Saccharomycopsis* to rely solely on Flo8 homodimers to direct flocculation. *S. fermentans FLO8* is one of only 24 genes encoding a poly-Q stretch. These poly-glutamines could additionally promote protein–protein interactions and thus in the case of *S. fermentans* Flo8 facilitate homodimerization (Perutz et al., 1994).

## Conclusion and Outlook

This work has generated new insight into the suitability of *S. fermentans* for industrial beverage fermentations. We found a similar telomere association of adhesin genes in *S. fermentans* as it is known for *FLO* genes in *S. cerevisiae*. *S. fermentans* harbor a large set of adhesins encoded by the *FAS* gene family, which could serve distinct purposes in flocculation or predation. Functionally, *S. fermentans* flocculation is phenotypically similar to flocculation in *S. cerevisiae* but apparently is regulated only by the Flo8 transcription factor and not by a heterodimer of Flo8 and Mss11 as in *S. cerevisiae* and *C. albicans*. As a next step the functional analysis of *Saccharomycopsis FLO8* for either flocculation or predation will be interesting to elucidate and also its ability to complement deletion of *ScFLO8* and/or *MSS11*. Introduction of *S. fermentans* as a novel non-conventional yeast in beer and wine fermentations may require selection of strains that are, e.g., adapted to stressful fermentation conditions and higher alcohol concentrations. In contrast to most brewer’s yeasts with limited sexual reproduction abilities, *S. fermentans* is a homothallic yeast that is amenable to yeast breeding. Thus, further characterization of this yeast and other species of the genus may lead to advanced molecular yeast breeding efforts to increase flavor diversity of alcoholic beverages in the future.

## Data Availability

All datasets analyzed for this study are included in the manuscript and the supplementary files or are available online.

## Author Contributions

BB, YK, and JW contributed to conception and design of the study. All authors contributed to experimental or bioinformatic analyses. JW analyzed and interpreted the data, introduced the term “telolog,” and wrote the manuscript draft. All authors contributed to manuscript revision.

## Conflict of Interest Statement

The authors declare that the research was conducted in the absence of any commercial or financial relationships that could be construed as a potential conflict of interest.

## Footnotes

1 https://www.yeastgenome.org/

2 https://www.ebi.ac.uk/Tools/pfa/radar/

3 http://www.candidagenome.org/

4 https://blast.ncbi.nlm.nih.gov/Blast.cgi

5 http://agd.unibas.ch

6 https://www.ncbi.nlm.nih.gov/Structure/bwrpsb/bwrpsb.cgi

## Supplementary Material

TABLE S1 Table providing sequence details of the *S. fermentans FAS* gene family indicating the length, number of internal repeats, closest relative within *S. fermentans* and the ORF and translated protein sequences of all 34 *FAS* genes.

FIGURE S1 Table indicating pairwise amino acid sequence identity between adhesins of the *S. fermentans* Fas family, the *S. cerevisiae* Flo family and the *C. albicans* Als family.

## References

[B1] Aslankoohi E. Herrera-Malaver B. Rezaei M. N. Steensels J. Courtin C. M. Verstrepen K. J. (2016). Non-conventional yeast strains increase the aroma complexity of bread. *PLoS One* 11:e0165126. 10.1371/journal.pone.0165126 27776154 PMC5077118

[B2] Bester M. C. Pretorius I. S. Bauer F. F. (2006). The regulation of *Saccharomyces cerevisiae* FLO gene expression and Ca2 + -dependent flocculation by Flo8p and Mss11p. *Curr. Genet.* 49 375–383. 10.1007/s00294-006-0068-z 16568252

[B3] Blomqvist J. Passoth V. (2015). Dekkera bruxellensis–spoilage yeast with biotechnological potential, and a model for yeast evolution, physiology and competitiveness. *FEMS Yeast Res.* 15:fov021. 10.1093/femsyr/fov021 25956542

[B4] Bony M. Thines-Sempoux D. Barre P. Blondin B. (1997). Localization and cell surface anchoring of the Saccharomyces cerevisiae flocculation protein Flo1p. *J. Bacteriol.* 179 4929–4936. 10.1128/jb.179.15.4929-4936.1997 9244284 PMC179343

[B5] Boulianne R. P. Liu Y. Aebi M. Lu B. C. Kues U. (2000). Fruiting body development in *Coprinus cinereus*: regulated expression of two galectins secreted by a non-classical pathway. *Microbiology* 146(Pt 8), 1841–1853. 10.1099/00221287-146-8-1841 10931889

[B6] Boyd W. C. Shapleigh E. (1954). Specific precipitating activity of plant agglutinins (Lectins). *Science* 119:419. 10.1126/science.119.3091.419 17842730

[B7] Brückner S. Mösch H. U. (2012). Choosing the right lifestyle: adhesion and development in *Saccharomyces cerevisiae.* *FEMS Microbiol. Rev.* 36 25–58. 10.1111/j.1574-6976.2011.00275.x 21521246

[B8] Bussey H. Kaback D. B. Zhong W. Vo D. T. Clark M. W. Fortin N. (1995). The nucleotide sequence of chromosome I from Saccharomyces cerevisiae. *Proc. Natl. Acad. Sci. U.S.A.* 92 3809–3813. 10.1073/pnas.92.9.3809 7731988 PMC42051

[B9] Cao F. Lane S. Raniga P. P. Lu Y. Zhou Z. Ramon K. (2006). The Flo8 transcription factor is essential for hyphal development and virulence in Candida albicans. *Mol. Biol. Cell* 17 295–307. 10.1091/mbc.e05-06-0502 16267276 PMC1345667

[B10] Capozzi V. Garofalo C. Chiriatti M. A. Grieco F. Spano G. (2015). Microbial terroir and food innovation: the case of yeast biodiversity in wine. *Microbiol. Res.* 181 75–83. 10.1016/j.micres.2015.10.005 26521127

[B11] Carro D. Garcia-Martinez J. Perez-Ortin J. E. Pina B. (2003). Structural characterization of chromosome I size variants from a natural yeast strain. *Yeast* 20 171–183. 10.1002/yea.955 12518320

[B12] Carroll E. Trinh T. N. Son H. Lee Y. W. Seo J. A. (2017). Comprehensive analysis of fungal diversity and enzyme activity in nuruk, a Korean fermenting starter, for acquiring useful fungi. *J. Microbiol.* 55 357–365. 10.1007/s12275-017-7114-z 28455587

[B13] Choo J. H. Hong C. P. Lim J. Y. Seo J. A. Kim Y. S. Lee D. W. (2016). Whole-genome de novo sequencing, combined with RNA-Seq analysis, reveals unique genome and physiological features of the amylolytic yeast *Saccharomycopsis fibuligera* and its interspecies hybrid. *Biotechnol. Biofuels* 9:246. 10.1186/s13068-016-0653-4 27872659 PMC5106798

[B14] Ciani M. Morales P. Comitini F. Tronchoni J. Canonico L. Curiel J. A. (2016). Non-conventional yeast species for lowering ethanol content of wines. *Front. Microbiol.* 7:642. 10.3389/fmicb.2016.00642 27199967 PMC4854890

[B15] Cullen P. J. (2015). The plate-washing assay: a simple test for filamentous growth in budding yeast. *Cold Spring Harb. Protoc.* 2015 168–171. 10.1101/pdb.prot085068 25646503 PMC4451083

[B16] De Filippis F. La Storia A. Blaiotta G. (2017). Monitoring the mycobiota during Greco di Tufo and aglianico wine fermentation by 18S rRNA gene sequencing. *Food Microbiol.* 63 117–122. 10.1016/j.fm.2016.11.010 28040157

[B17] De Groot P. W. Bader O. De Boer A. D. Weig M. Chauhan N. (2013). Adhesins in human fungal pathogens: glue with plenty of stick. *Eukaryot. Cell* 12 470–481. 10.1128/EC.00364-12 23397570 PMC3623432

[B18] Dranginis A. M. Rauceo J. M. Coronado J. E. Lipke P. N. (2007). A biochemical guide to yeast adhesins: glycoproteins for social and antisocial occasions. *Microbiol. Mol. Biol. Rev.* 71 282–294. 10.1128/MMBR.00037-06 17554046 PMC1899881

[B19] Erdman S. Lin L. Malczynski M. Snyder M. (1998). Pheromone-regulated genes required for yeast mating differentiation. *J. Cell Biol.* 140 461–483. 10.1083/jcb.140.3.461 9456310 PMC2140177

[B20] Farh M. E. Cho Y. Lim J. Y. Seo J. A. (2017). A diversity study of *Saccharomycopsis fibuligera* in rice wine starter nuruk, reveals the evolutionary process associated with its interspecies hybrid. *J. Microbiol.* 55 337–343. 10.1007/s12275-017-7115-y 28455588

[B21] Fichtner L. Schulze F. Braus G. H. (2007). Differential Flo8p-dependent regulation of FLO1 and FLO11 for cell-cell and cell-substrate adherence of S. *cerevisiae* S288c. *Mol. Microbiol.* 66 1276–1289. 10.1111/j.1365-2958.2007.06014.x 18001350 PMC2780560

[B22] Finn R. D. Miller B. L. Clements J. Bateman A. (2014). iPfam: a database of protein family and domain interactions found in the Protein Data Bank. *Nucleic Acids Res.* 42 D364–D373. 10.1093/nar/gkt1210 24297255 PMC3965099

[B23] Gabaldon T. Martin T. Marcet-Houben M. Durrens P. Bolotin-Fukuhara M. Lespinet O. (2013). Comparative genomics of emerging pathogens in the Candida glabrata clade. *BMC Genomics* 14:623. 10.1186/1471-2164-14-623 24034898 PMC3847288

[B24] Goossens K. Willaert R. (2010). Flocculation protein structure and cell-cell adhesion mechanism in Saccharomyces cerevisiae. *Biotechnol. Lett.* 32 1571–1585. 10.1007/s10529-010-0352-3 20640875

[B25] Gordon J. L. Byrne K. P. Wolfe K. H. (2009). Additions, losses, and rearrangements on the evolutionary route from a reconstructed ancestor to the modern Saccharomyces cerevisiae genome. *PLoS Genet.* 5:e1000485. 10.1371/journal.pgen.1000485 19436716 PMC2675101

[B26] Halme A. Bumgarner S. Styles C. Fink G. R. (2004). Genetic and epigenetic regulation of the FLO gene family generates cell-surface variation in yeast. *Cell* 116 405–415. 10.1016/S0092-8674(04)00118-7 15016375

[B27] Hassan M. A. Rouf R. Tiralongo E. May T. W. Tiralongo J. (2015). Mushroom lectins: specificity, structure and bioactivity relevant to human disease. *Int. J. Mol. Sci.* 16 7802–7838. 10.3390/ijms16047802 25856678 PMC4425051

[B28] Hesselbart A. Junker K. Wendland J. (2018). Draft genome sequence of *Saccharomycopsis fermentans* CBS 7830, a predacious yeast belonging to the *Saccharomycetales*. *Genome Announc* 6:e01445-17. 10.1128/genomeA.01445-17 29326220 PMC5764944

[B29] Hirabayashi J. Tateno H. Shikanai T. Aoki-Kinoshita K. F. Narimatsu H. (2015). The lectin frontier database (LfDB), and data generation based on frontal affinity chromatography. *Molecules* 20 951–973. 10.3390/molecules20010951 25580689 PMC6272529

[B30] Hoyer L. L. Cota E. (2016). Candida albicans agglutinin-like sequence (Als) family vignettes: a review of als protein structure and function. *Front. Microbiol.* 7:280. 10.3389/fmicb.2016.00280 27014205 PMC4791367

[B31] Illeghems K. De Vuyst L. Papalexandratou Z. Weckx S. (2012). Phylogenetic analysis of a spontaneous cocoa bean fermentation metagenome reveals new insights into its bacterial and fungal community diversity. *PLoS One* 7:e38040. 10.1371/journal.pone.0038040 22666442 PMC3362557

[B32] Jentoft N. (1990). Why are proteins O-glycosylated? *Trends Biochem. Sci.* 15 291–294. 2204153 10.1016/0968-0004(90)90014-3

[B33] Kim K. H. Hong S. K. Hwang K. Y. Kim E. E. (2014). Structure of mouse muskelin discoidin domain and biochemical characterization of its self-association. *Acta Crystallogr. D. Biol. Crystallogr.* 70 2863–2874. 10.1107/S139900471401894X 25372678

[B34] Kim M. H. Cooper D. R. Oleksy A. Devedjiev Y. Derewenda U. Reiner O. (2004). The structure of the N-terminal domain of the product of the lissencephaly gene Lis1 and its functional implications. *Structure* 12 987–998. 10.1016/j.str.2004.03.024 15274919

[B35] Klemm P. Schembri M. A. (2000). Fimbriae-assisted bacterial surface display of heterologous peptides. *Int. J. Med. Microbiol.* 290 215–221. 10.1016/S1438-4221(00)80118-6 10959723 PMC7129006

[B36] Kobayashi O. Suda H. Ohtani T. Sone H. (1996). Molecular cloning and analysis of the dominant flocculation gene FLO8 from *Saccharomyces cerevisiae*. *Mol. Gen. Genet.* 251 707–715. 8757402 10.1007/BF02174120

[B37] Kurtzman C. P. Robnett C. J. (2013). Relationships among genera of the *Saccharomycotina* (Ascomycota) from multigene phylogenetic analysis of type species. *FEMS Yeast Res.* 13 23–33. 10.1111/1567-1364.12006 22978764

[B38] Lachance M. A. Pang W. M. (1997). Predacious yeasts. *Yeast* 13 225–232. 10.1002/(SICI)1097-0061(19970315)13:3<225::AID-YEA87>3.0.CO;2-I 9090051

[B39] Lachance M. A. Pupovac-Velikonja A. Natarajan S. Schlag-Edler B. (2000). Nutrition and phylogeny of predacious yeasts. *Can. J. Microbiol.* 46 495–505. 10.1139/w00-021 10913970

[B40] Lipke P. N. (2018). What we do not know about fungal cell adhesion molecules. *J. Fungi* 4:E59. 10.3390/jof4020059 29772751 PMC6023273

[B41] Liti G. Louis E. J. (2005). Yeast evolution and comparative genomics. *Annu. Rev. Microbiol.* 59 135–153. 10.1146/annurev.micro.59.030804.121400 15877535

[B42] Lo W. S. Dranginis A. M. (1998). The cell surface flocculin Flo11 is required for pseudohyphae formation and invasion by *Saccharomyces cerevisiae*. *Mol. Biol. Cell* 9 161–171. 10.1091/mbc.9.1.161 9436998 PMC25236

[B43] Lopez-Fuentes E. Gutierrez-Escobedo G. Timmermans B. Van Dijck P. De Las Penas A. Castano I. (2018). Candida glabrata’s genome plasticity confers a unique pattern of expressed cell wall proteins. *J. Fungi* 4:E67. 10.3390/jof4020067 29874814 PMC6023349

[B44] Luo Z. Van Vuuren H. J. (2009). Functional analyses of PAU genes in *Saccharomyces cerevisiae*. *Microbiology* 155 4036–4049. 10.1099/mic.0.030726-0 19762443

[B45] Nag D. K. Suri M. Stenson E. K. (2004). Both CAG repeats and inverted DNA repeats stimulate spontaneous unequal sister-chromatid exchange in *Saccharomyces cerevisiae*. *Nucleic Acids Res.* 32 5677–5684. 10.1093/nar/gkh901 15494455 PMC524308

[B46] Naglik J. R. Challacombe S. J. Hube B. (2003). Candida albicans secreted aspartyl proteinases in virulence and pathogenesis. *Microbiol. Mol. Biol. Rev.* 67 400–428. 10.1128/MMBR.67.3.400-428.2003 12966142 PMC193873

[B47] Ouoba L. I. Kando C. Parkouda C. Sawadogo-Lingani H. Diawara B. Sutherland J. P. (2012). The microbiology of Bandji, palm wine of *Borassus akeassii* from Burkina faso: identification and genotypic diversity of yeasts, lactic acid and acetic acid bacteria. *J. Appl. Microbiol.* 113 1428–1441. 10.1111/jam.12014 22979949

[B48] Perutz M. F. Johnson T. Suzuki M. Finch J. T. (1994). Glutamine repeats as polar zippers: their possible role in inherited neurodegenerative diseases. *Proc. Natl. Acad. Sci. U.S.A.* 91 5355–5358. 10.1073/pnas.91.12.5355 8202492 PMC43993

[B49] Peter J. De Chiara M. Friedrich A. Yue J. X. Pflieger D. Bergstrom A. (2018). Genome evolution across 1,011 *Saccharomyces cerevisiae* isolates. *Nature* 556 339–344. 10.1038/s41586-018-0030-5 29643504 PMC6784862

[B50] Reynolds T. B. Fink G. R. (2001). Bakers’ yeast, a model for fungal biofilm formation. *Science* 291 878–881. 10.1126/science.291.5505.878 11157168

[B51] Rigden D. J. Mello L. V. Galperin M. Y. (2004). The PA14 domain, a conserved all-beta domain in bacterial toxins, enzymes, adhesins and signaling molecules. *Trends Biochem. Sci.* 29 335–339. 10.1016/j.tibs.2004.05.002 15236739

[B52] Rossouw D. Bauer F. F. (2016). Exploring the phenotypic space of non-Saccharomyces wine yeast biodiversity. *Food Microbiol.* 55 32–46. 10.1016/j.fm.2015.11.017 26742614

[B53] Schifferdecker A. J. Dashko S. Ishchuk O. P. Piskur J. (2014). The wine and beer yeast *Dekkera bruxellensis*. *Yeast* 31 323–332. 10.1002/yea.3023 24932634 PMC4257070

[B54] Schild L. Heyken A. De Groot P. W. Hiller E. Mock M. De Koster C. (2011). Proteolytic cleavage of covalently linked cell wall proteins by Candida albicans Sap9 and Sap10. *Eukaryot. Cell* 10 98–109. 10.1128/EC.00210-10 21097664 PMC3019796

[B55] Seidl V. Huemer B. Seiboth B. Kubicek C. P. (2005). A complete survey of *Trichoderma chitinases* reveals three distinct subgroups of family 18 chitinases. *FEBS J.* 272 5923–5939. 10.1111/j.1742-4658.2005.04994.x 16279955

[B56] Sharon N. Lis H. (2004). History of lectins: from hemagglutinins to biological recognition molecules. *Glycobiology* 14 53R–62R. 10.1093/glycob/cwh122 15229195

[B57] Shrestha B. Guragain B. Sridhar V. V. (2014). Involvement of co-repressor LUH and the adapter proteins SLK1 and SLK2 in the regulation of abiotic stress response genes in Arabidopsis. *BMC Plant Biol.* 14:54. 10.1186/1471-2229-14-54 24564815 PMC4015341

[B58] Silva C. F. Batista L. R. Abreu L. M. Dias E. S. Schwan R. F. (2008). Succession of bacterial and fungal communities during natural coffee (*Coffea arabica*) fermentation. *Food Microbiol.* 25 951–957. 10.1016/j.fm.2008.07.003 18954729

[B59] Snauwaert I. Roels S. P. Van Nieuwerburg F. Van Landschoot A. De Vuyst L. Vandamme P. (2016). Microbial diversity and metabolite composition of Belgian red-brown acidic ales. *Int. J. Food Microbiol.* 221 1–11. 10.1016/j.ijfoodmicro.2015.12.009 26802571

[B60] Soares E. V. (2011). Flocculation in *Saccharomyces cerevisiae*: a review. *J. Appl. Microbiol.* 110 1–18. 10.1111/j.1365-2672.2010.04897.x 21114594

[B61] Steensels J. Daenen L. Malcorps P. Derdelinckx G. Verachtert H. Verstrepen K. J. (2015). Brettanomyces yeasts–from spoilage organisms to valuable contributors to industrial fermentations. *Int. J. Food Microbiol.* 206 24–38. 10.1016/j.ijfoodmicro.2015.04.005 25916511

[B62] Steenwyk J. Rokas A. (2017). Extensive copy number variation in fermentation-related genes among *Saccharomyces cerevisiae* wine strains. *G3* 7 1475–1485. 10.1534/g3.117.040105 28292787 PMC5427499

[B63] Sternes P. R. Lee D. Kutyna D. R. Borneman A. R. (2017). A combined meta-barcoding and shotgun metagenomic analysis of spontaneous wine fermentation. *Gigascience* 6 1–10. 10.1093/gigascience/gix040 28595314 PMC5570097

[B64] Stewart G. G. (2018). Yeast flocculation—sedimentation and flotation. *Fermentation* 4:28 10.3390/fermentation4020028

[B65] Stratford M. (1989). Evidence for two mechanisms of flocculation in *Saccharomyces cerevisiae*. *Yeast* 5 S441–S445. 2665372

[B66] Su C. Li Y. Lu Y. Chen J. (2009). Mss11, a transcriptional activator, is required for hyphal development in Candida albicans. *Eukaryot. Cell* 8 1780–1791. 10.1128/EC.00190-09 19734367 PMC2772397

[B67] Sui X. Yan L. Jiang Y. Y. (2017). The vaccines and antibodies associated with Als3p for treatment of Candida albicans infections. *Vaccine* 35 5786–5793. 10.1016/j.vaccine.2017.08.082 28911903

[B68] Teunissen A. W. Steensma H. Y. (1995). Review: the dominant flocculation genes of *Saccharomyces cerevisiae* constitute a new subtelomeric gene family. *Yeast* 11 1001–1013. 10.1002/yea.320111102 7502576

[B69] Teunissen A. W. Van Den Berg J. A. Steensma H. Y. (1995). Transcriptional regulation of flocculation genes in Saccharomyces cerevisiae. *Yeast* 11 435–446. 10.1002/yea.320110506 7597847

[B70] Urso R. Rantsiou K. Dolci P. Rolle L. Comi G. Cocolin L. (2008). Yeast biodiversity and dynamics during sweet wine production as determined by molecular methods. *FEMS Yeast Res.* 8 1053–1062. 10.1111/j.1567-1364.2008.00364.x 18341578

[B71] Verstrepen K. J. Derdelinckx G. Verachtert H. Delvaux F. R. (2003). Yeast flocculation: what brewers should know. *Appl. Microbiol. Biotechnol.* 61 197–205. 10.1007/s00253-002-1200-8 12698276

[B72] Verstrepen K. J. Fink G. R. (2009). Genetic and epigenetic mechanisms underlying cell-surface variability in protozoa and fungi. *Annu. Rev. Genet.* 43 1–24. 10.1146/annurev-genet-102108-134156 19640229

[B73] Verstrepen K. J. Jansen A. Lewitter F. Fink G. R. (2005). Intragenic tandem repeats generate functional variability. *Nat. Genet.* 37 986–990. 10.1038/ng1618 16086015 PMC1462868

[B74] Verstrepen K. J. Klis F. M. (2006). Flocculation, adhesion and biofilm formation in yeasts. *Mol. Microbiol.* 60 5–15. 10.1111/j.1365-2958.2006.05072.x 16556216

[B75] Wälti M. A. Thore S. Aebi M. Kunzler M. (2008). Crystal structure of the putative carbohydrate recognition domain of human galectin-related protein. *Proteins* 72 804–808. 10.1002/prot.22078 18433051

[B76] Wu R. Yu M. Liu X. Meng L. Wang Q. Xue Y. (2015). Changes in flavour and microbial diversity during natural fermentation of suan-cai, a traditional food made in Northeast China. *Int. J. Food Microbiol.* 211 23–31. 10.1016/j.ijfoodmicro.2015.06.028 26159472

