# Supplementary figures and images for: Expansion of a Telomeric FLO/ALS-Like Sequence Gene Family in Saccharomycopsis fermentans
Source: Front Genet. 2018 Nov 13;9:536. doi: 10.3389/fgene.2018.00536 (PMC6277891; doi:10.3389/fgene.2018.00536)

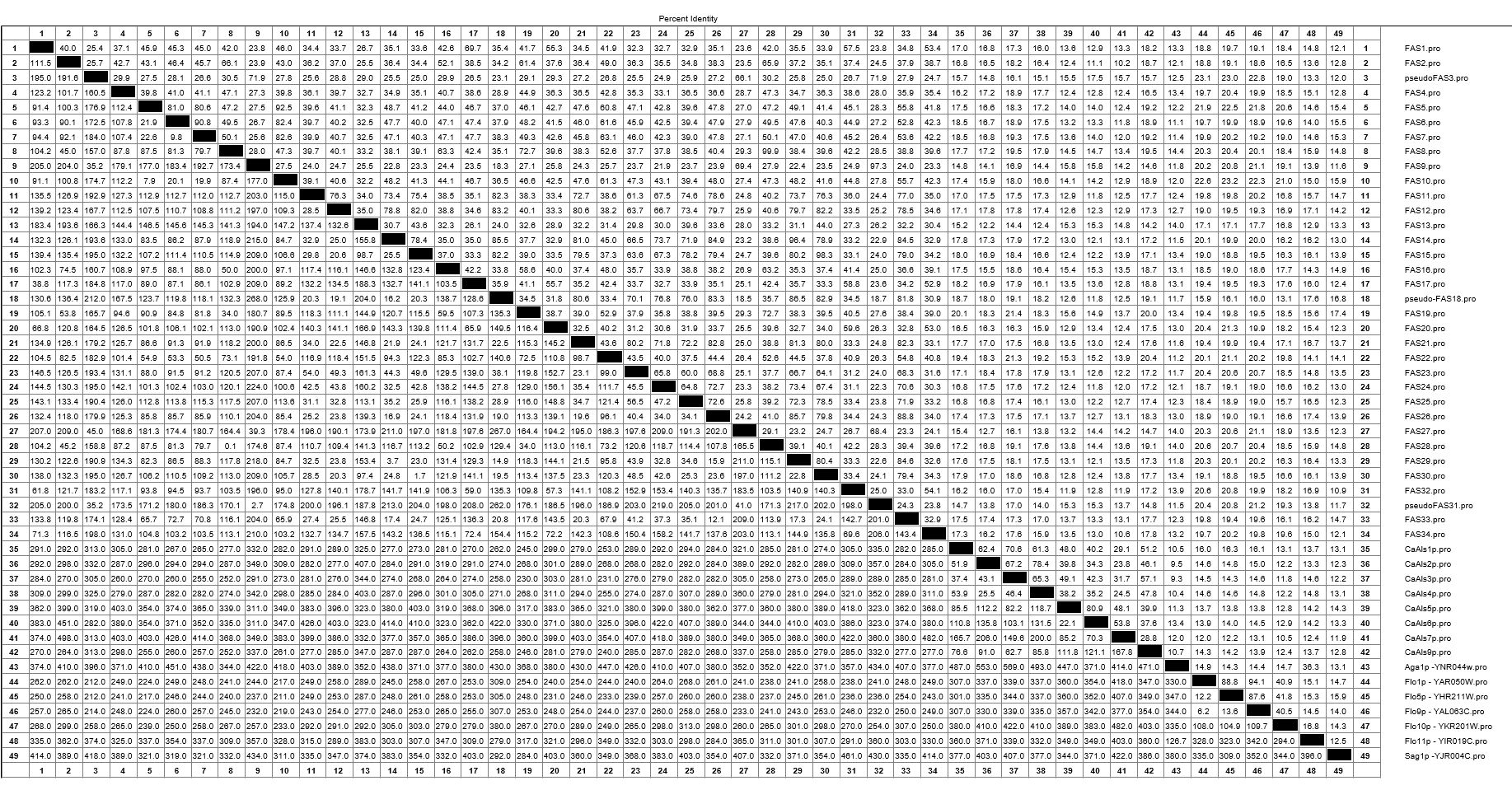

Supplement: FIGURE S1 — Table indicating pairwise amino acid sequence identity between adhesins of the S. fermentans Fas family, the S. cerevisiae Flo family and the C. albicans Als family. [file Image_1.JPEG]
